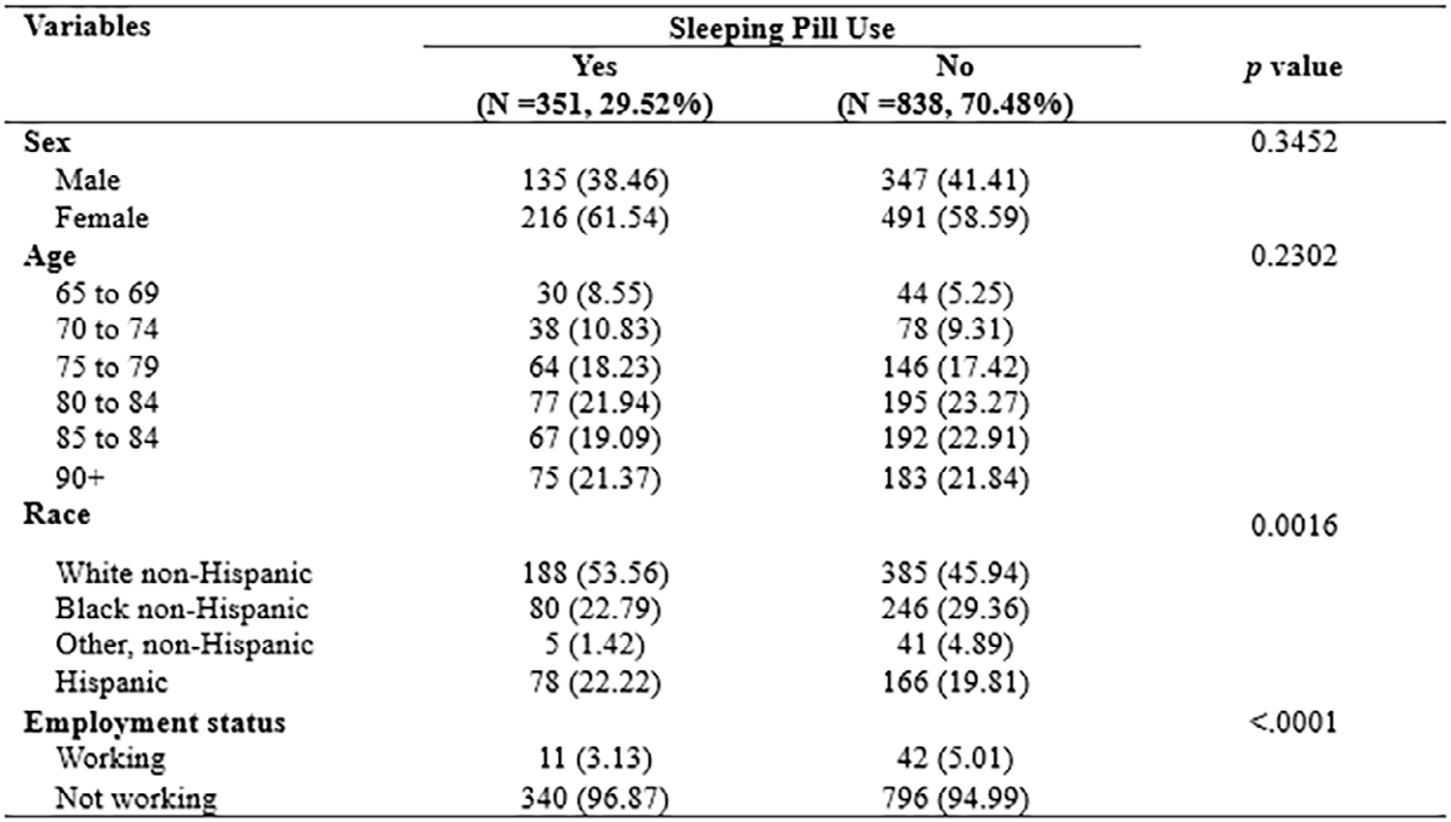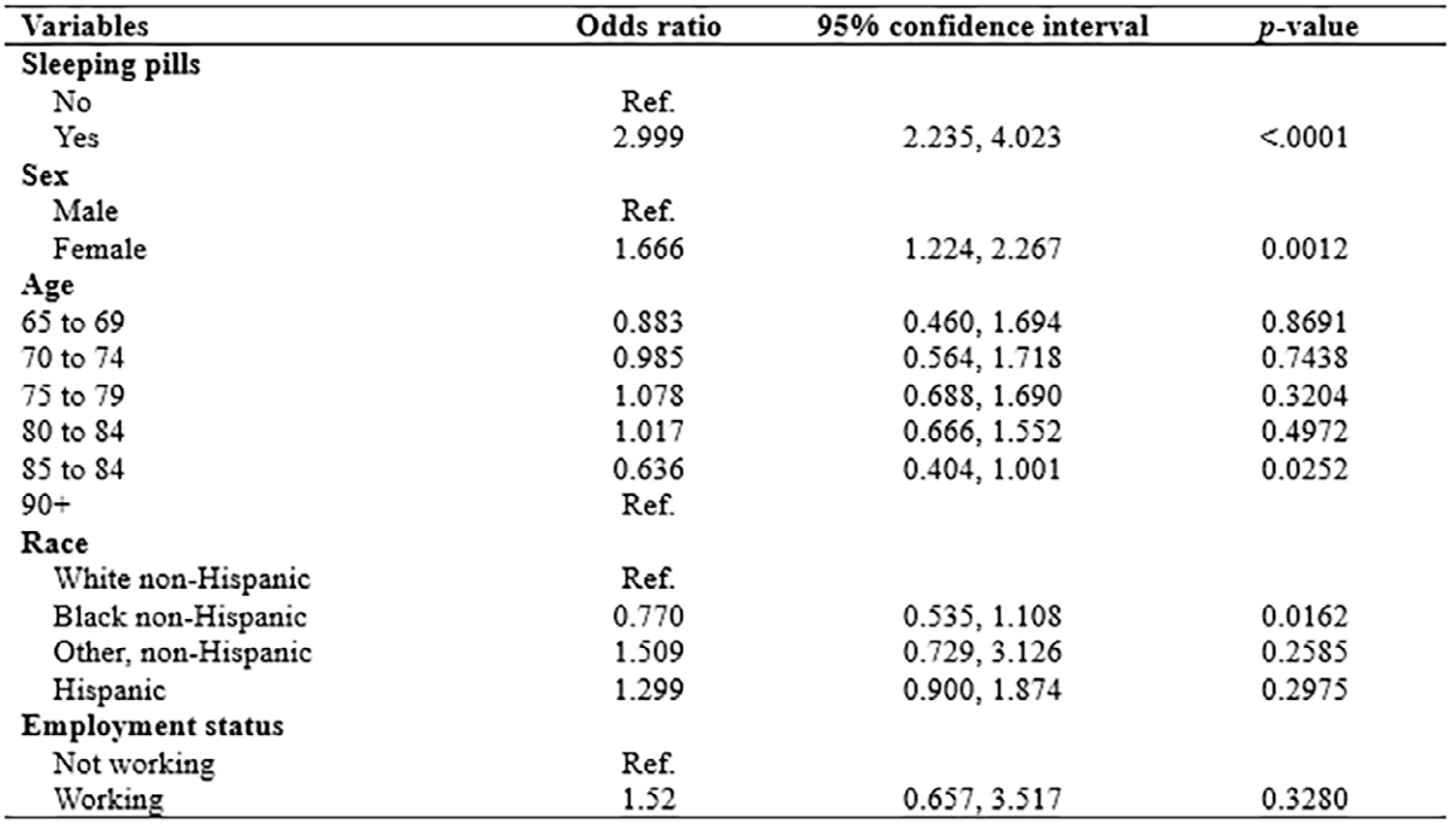# Association of Sleeping Pills on Anxiety Symptoms in Dementia and Borderline Groups: Insights from the 2022 National Health and Aging Trends Study

**DOI:** 10.1002/alz.088730

**Published:** 2025-01-03

**Authors:** Sanghee Yoo, Ickpyo Hong

**Affiliations:** ^1^ Yonsei university, Wonju, Gangwon‐do Korea, Republic of (South); ^2^ Yonsei University, Wonju, Gangwon‐do Korea, Republic of (South)

## Abstract

**Background:**

Old adults' sleep patterns change during the aging process^1^. Among old adults, 40% of individuals experience insufficient sleep and complain of sleeping disorder, including a decline in the quality of sleep^2^. Patients with dementia also experience sleep disorders, and the most common intervention for this is pharmacotherapy^3,4^. This study aims to investigate the relationship between anxiety and sleeping pills in patients with dementia and borderline groups.

**Method:**

In this study, the 2022National Health and Aging Trends Study was used to examine the association between anxiety symptoms and sleeping pill use in patients with dementia and borderline groups. The analysis was conducted using a multiple regression model by accounting for demographics.

**Result:**

This study analyzed a sample of 1,189 and there were 351 (29.52%) patients with dementia and borderline groups who reported taking sleeping pills. As a result of multiple regression analysis, the group that took drugs for sleep showed a significantly higher risk of anxiety symptoms than the group that did not take drugs (odds ratio [OR] 2.999; 95% confidence interval [CI] 2.235, 4.023). Compared to men, the risk of showing anxiety symptoms was significantly higher in women (OR 1.666; 95% CI 1.224, 2.267). Compared to whites, blacks had a significantly lower risk of anxiety (OR 0.770; 95%CI 0.535, 1.108).

**Conclusion:**

This study showed a strong association between sleep medication use and anxiety symptoms in American patients with dementia and a borderline group. This suggests the need for treatment that addresses the mental health of dementia and borderline groups who experience sleep disorders.